# Automated Guava Disease Detection Using Transfer Learning With ResNet‐101

**DOI:** 10.1002/fsn3.71348

**Published:** 2025-12-21

**Authors:** Muhammad Ahmed, Fahad Ahmed, Naila Sammar Naz, Tehseen Mazhar, Muhammad Adnan Khan, Muhammad Amir Khan, Amel Ksibi, Mohamed Abbas

**Affiliations:** ^1^ School of Computer Science National College of Business Administration and Economics Lahore Pakistan; ^2^ Department of Software, Faculty of Artificial Intelligence and Software Gachon University Seongnam‐si Republic of Korea; ^3^ Faculty of Computer and Mathematical Sciences Universiti Teknologi MARA 40450 Shah Alam Selangor Malaysia; ^4^ Department of Information Systems, College of Computer and Information Sciences Princess Nourah Bint Abdulrahman University Riyadh Saudi Arabia; ^5^ Electrical Engineering Department, College of Engineering King Khalid University Abha Saudi Arabia; ^6^ Department of Physics, Saveetha School of Engineering Saveetha Institute of Medical and Technical Sciences (SIMATS) India

**Keywords:** deep learning, explainable AI, guava disease detection, ResNet‐101, transfer learning

## Abstract

The old method of identifying diseases, which farmers used by manually inspecting their farms, is ineffective because it is time‐consuming, prone to human error, and cannot be applied to a wide agricultural territory. Sustainable agriculture thus requires automated disease detection that provides accurate results to meet the increasing demand for this technology. This paper explores the use of deep learning (DL) and transfer learning (TL) using ResNet‐101 to advance the guava disease detection. The image information is raw, and the image information is directly given to ResNet‐101, which can recognize complex patterns without manually extracting features and hence creating an effective and accurate classification method. To make the sample size bigger and more balanced, data augmentation was applied to the original collection of 3784 images and resulted in the generation of 4632 images in equal proportions in three categories of health condition: Anthracnose, Fruit Fly, and Healthy guavas. The balanced dataset was separated into three parts: the training, the validation, and the test parts that consisted of 80%, 10%, and 10%, respectively, to make sure that the model is well trained and tested. The preprocessing of the data was also done by normalization and resizing methods, which improved the performance of the model. The accuracy of the proposed model was found to be impressive, with 98.48% being the percentage of accuracy in the classification of the guava disease, and it is exhaustively tested in conserving nine main measures, which are accuracy; misclassification rate, specificity, recall, precision, negative predictive value (NPV), false positive rate (FPR), false negative rate (FNR), and F1 score. In order to provide interpretability, fairness, and transparency, Gradient‐weighted Class Activation Mapping (Grad‐CAM) visualization was used, generating heatmaps that reveal the diseased areas and which also make sure that the network concentrates on the real areas of infection. It is an artificial intelligence (AI) technology that provides a better identification of plant diseases and is a viable solution in large‐scale agriculture.

## Graphical abstract

A DL model based on ResNet‐101, which is a state‐of‐the‐art CNN enhanced through TL implementation. The method enables the model to utilize pre‐trained weights, which are adapted for detecting guava diseases while reducing training duration and improving results. The model detects and organizes three types of guava conditions: Anthracnose, fruit fly, and healthy Guava. When farmers use automatic detection tools, the system provides them with an accurate monitoring system to observe crop health, enabling them to make prompt decisions for better loss prevention. A comprehensive data augmentation process was implemented to enhance the data and mitigate the initial class imbalance. The sample size was increased to 4632 by using methods such as rotation, flipping, and minor variations in brightness, which resulted in an even distribution between the samples of Anthracnose, Fruit Fly, and Healthy guava. This more varied and balanced dataset introduced additional visual patterns to the model, allowing it to generalize and further reduce the likelihood of overfitting, and ultimately improving classification accuracy and reliability. The implemented ResNet‐101 model achieves an exceptional accuracy of 98.48%, outperforming all models discussed in the cited literature [8, 9]. The accuracy of 98.48% shows that our methodology provides successful performance in diagnosing guava diseases. The model was evaluated based on its accuracy determination, as well as several performance measures, including misclassification rate, specificity, NPV, FPR, and FNR. Testing according to these measures can give a complete picture of the practical reliability and usefulness of the tested model. Grad‐CAM is used in the model to simplify the predictions made by the DL. This XAI technique creates heatmaps that visually indicate the most important regions of the image that will influence the decision made by the model. Grad‐CAM makes predictions transparent and identifies points of infection or pest damage that motivate individual predictions, which in turn enhance the level of trust in the model for real‐life settings, thereby vindicating the effectiveness of the model. Architecture of the proposed model.

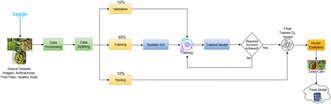

## 1 Introduction

One of the most pressing problems of our time is food production, given the fast‐growing world population. It is forecast that the world's food consumption will have quadrupled by 2050. Food production needs a more efficient and sustainable environment to maximize plant yield (Hunter et al. 2017; Mirvakhabova et al. 2018). Belonging to the Myrtaceae family, the guava is among the most significant plants in the world. Originally from the tropical regions of the Americas, the guava was introduced to Portugal in the early 17th century. Many tropical and nontropical countries appreciate it, including Bangladesh, Pakistan, India, Brazil, and Cuba (Almadhor et al. 2021). Among the essential nutrients in guava are phosphorus, calcium, and nicotinic acid. Guavas can also develop dastur, another condition caused by dry rot. In guava production, the consequences of diseases like these lead to the depletion of both ecological and economic resources (Shadrin et al. 2020). Any kind of depletion of fresh air, water, energy, or land is regarded as an “environmental loss.” This results in a manufacturing shortfall, leading to an economic loss. Located in the Asia‐Pacific area, Pakistan derives most of its The Gross Domestic Product (GDP) from farming. GDP is one measure used to gauge the significance of Pakistan's agricultural industry. The country of Pakistan contributes to the yearly GDP of Afghanistan by 50%. Guava is a farming crop that is grown in most producing nations. Pakistan produces 1,784,300 short tons annually and is position four in the world. Timely diagnosis of the guava diseases will be determined by proper detection because when the diseases are not correctly diagnosed, their yield can considerably below. Manual observation is labor intensive although it may give a false result.

**FIGURE 1:**
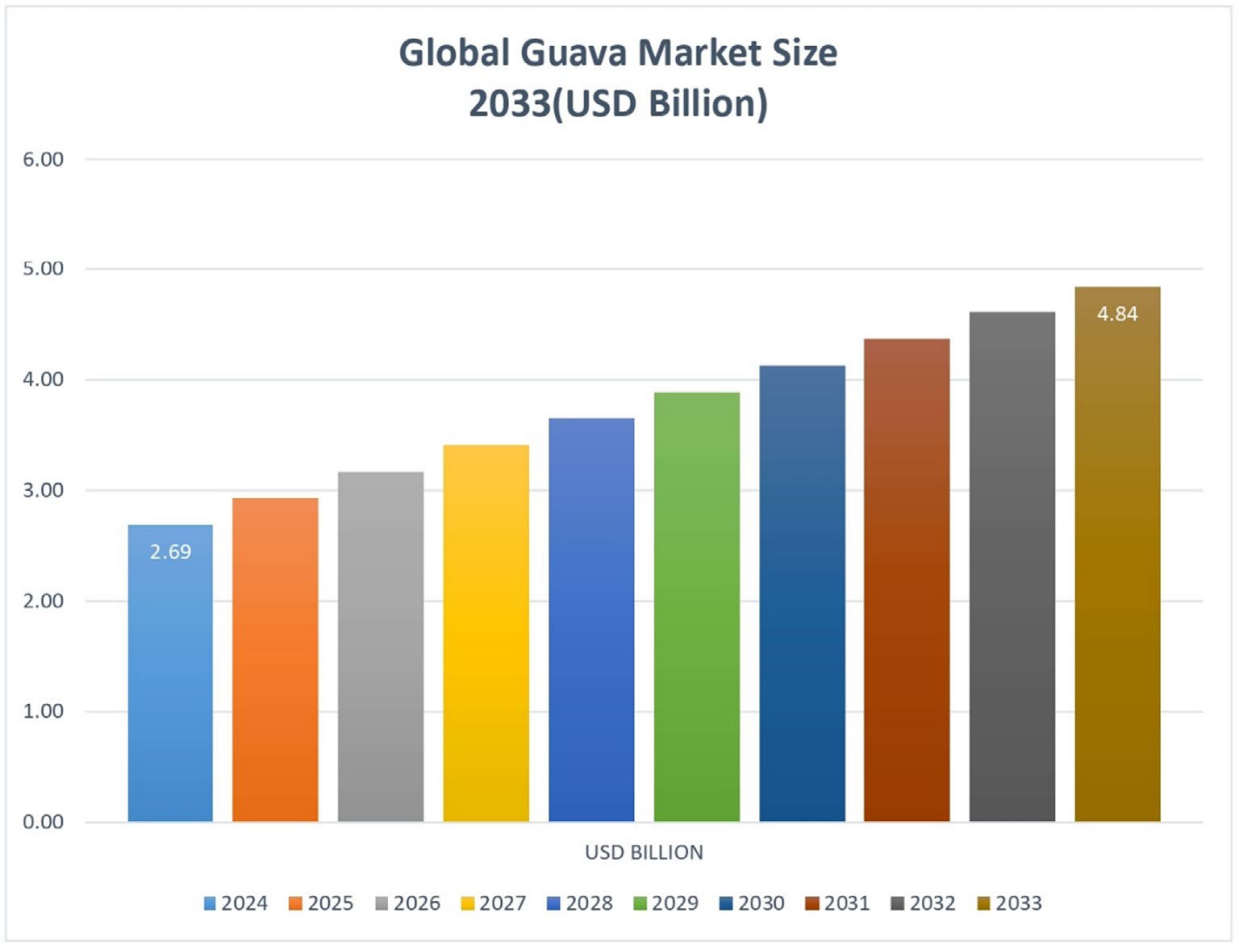
Global Guava Market Growth (2024–2033): Market Size in USD Billion (https://www.businessresearchinsights.com/market‐reports/guava‐market‐115950).

*Psidium guajava* , also referred to as Guava, tropical and subtropical fruit, has a very useful nutritional essence and ensures economic value since it is versatile in terms of scientific uses. Guava has been referred to as the poor man's apple since it is rich in numerous antioxidants, vitamin C, and other essential bioactive compounds that offer numerous health benefits to the consumers. Guava fruits and leaves were utilized in conventional herbal medicine in the treatment of digestive disorders, treatment of wounds, and in the fight against inflammation. Colonial production methods convert the perishable guavas into juice products, spreads, and powders made of juice products, which increases their usability (Gupta et al. 2018). The global market of guava has developed as indicated in Figure 1.

The world market of guava is projected to exceed 4.84 billion in the year 2033 with a CAGR of 7.5 in 2024 when it is anticipated that the market will be 2.69 billion. This is due to the fact that the market growth is attributed to more consumers buying the guava products and their by‐products, as they get to know about the health benefits (https://www.businessresearchinsights.com/market‐reports/guava‐market‐115950). Growth of the guavas is found in the subtropical regions which span across the whole of Asia, Latin America, and Africa and the agricultural activities in India, Mexico, and Pakistan are the major supporters. The emerging consumer trend in the market to consume fresh guava and its processed products in terms of juices and jam proves both the economic importance of guava and its growing popularity across the world market (Merida and Palmateer 2006).

Guava agriculture poses a serious challenge to effective production since the presence of diseases and pests remains a challenge to the production. Infestation as well as anthracnose disease caused by Colletotrichum gloeosporioides are the most significant factors in guava cultivation, which results in reduced yield and poor quality (Almadhor et al. 2021). This makes it challenging to identify the problems by most farmers who are working in the developing countries since they do not have resources and know how to observe them on time. The world population is rising, and agricultural land is reducing, and there is the need to take urgent measures to boost the production levels of guava without making massive losses. Inspection of disease in guavas manually will not be efficient in large farms since the process involves massive work, consumes a significant portion of time, and yields poor results. The immediate need is a computerized program that will precisely analyze guava images to determine the presence of the disease. This automated system would help the farmers to manage their crops in a better way so that they could yield more quality guavas to fulfill the needs of the market.

Machine learning (ML) has been applied in the agricultural industry since ancient times. The handcrafted feature extraction process is used to choose manually determined image features, including color, texture, and shape due to the skills of experts in the domain. The manual feature extraction technique is effective under certain circumstances, and yet it takes a long time effort by both the professional personnel and thorough knowledge of the sector. Manual feature identification, which is followed by extraction, is time‐consuming to humans and leads to a number of human errors in the case of large datasets. The use of conventional ML methods has challenges with complex or high‐dimensional datasets such as plant imagery with large deviations and subtle disease symptoms. This practice makes the models nongeneralizable, and thus less effective in real‐world settings (Tripathi et al. 2023).

Convolutional neural networks (CNN) changed the domain of image analysis as the corresponding methods of the DL did not presuppose the necessity of human feature extraction. Recent DL architecture as ResNet101 has shown the ability to extract relevant features automatically on original works, such as images, without being manually supervised. DL models are effective in the extraction of hierarchical features automatically and thus, they are useful in identifying rich patterns in data particularly with the discovery of guava crop diseases. These models are able to process downscaled image datasets very fast and hence they are applicable in handling the large amount of agricultural image data. The accuracy of the DL models is beyond any other traditional ML models as well as the capability of generalizing data. They are able to differentiate between healthy and diseased plant variations when they are trained on large and varied datasets, not considering the environmental conditions like light and the angle (Mostafa et al. 2021).

DL is the ideal disease detection technique since it improves the speed and accuracy of disease detection in aspects that will deal with the issue of guava disease identification. Drivable DL models allow the development of long‐lasting automated systems to assist farmers in identifying crop threats at an early stage during crop monitoring activities. Such systems would also remove human inspections that would result in improved disease management due to enhanced decision‐making talents. DL demonstrates that it is better than traditional ML since it offers superior performance in operational efficiency and accuracy at the same time with great scalability; therefore, it is an essential component of the process of detecting agricultural diseases.

DL models are considered to be black boxes, something that limits the transparency, interpretability, and nonbiasness. In case of overcoming this limitation, the use of the Grad‐CAM method of explainable AI (XAI) was used to demonstrate the most important parts of an image that influence the prediction and thus obtain a more accurate understanding of how the model makes a decision.

The contributions of the study are

- A DL model based on ResNet‐101, which is a state‐of‐the‐art CNN enhanced through TL implementation. The method enables the model to utilize pre‐trained weights, which are adapted for detecting guava diseases while reducing training duration and improving results.
- The model detects and organizes three types of guava conditions: Anthracnose, fruit fly, and healthy Guava. When farmers use automatic detection tools, the system provides them with an accurate monitoring system to observe crop health, enabling them to make prompt decisions for better loss prevention.
- Fully implemented data augmentation scheme was undertaken to strengthen the data and overcome the first imbalance of classes. 4632 was the result of the methods of rotation, flipping and slight changes of the brightness and led to the even distributions of the amounts of the samples of Anthracnose, Fruit Fly and Healthy guava. This more diversified and balanced data contributed more visual patterns to the model to enable it to generalize and further minimize the chances of overfitting and eventually enhancing the classification accuracy and reliability.
- The implemented ResNet‐101 model achieves an exceptional accuracy of 98.48%, outperforming all models discussed in the cited literature (Mostafa et al. 2021; Kilci and Koklu 2024). The accuracy of 98.48% shows that our methodology provides successful performance in diagnosing guava diseases.
- The model was evaluated based on its accuracy determination, as well as several performance measures, including misclassification rate, specificity, NPV, FPR, and FNR. Testing according to these measures can give a complete picture of the practical reliability and usefulness of the tested model.
- Grad‐CAM is used in the model to simplify the predictions made by the DL. This XAI technique creates heatmaps that visually indicate the most important regions of the image that will influence the decision made by the model. Grad‐CAM makes predictions transparent and identifies points of infection or pest damage that motivate individual predictions, which in turn enhance the level of trust in the model for real‐life settings, thereby vindicating the effectiveness of the model.

The paper is structured as follows: Section 1 indicating the introduction. Section 2 gives the corresponding work section of the present paper, which is the description of earlier methodologies of detecting diseases. Section 3 outlines the research information of the Proposed Methodology by introducing the data set and explaining the model setup. Section 4 of the study outlines the process of simulations and experimental results. In Section 5, the results have been discussed in detail, covering the strengths, limitations, ethical considerations, and the practical aspects of deployment of the model. Last, Section 6 is the conclusion and future works, which will summarize the findings in general and give a suggestion on the direction of the research.

## 2 Related Work

The impending rapid technological developments make it possible to detect plant diseases in a much better way compared to the manual ways used to detect them through the hybrid DL that has substituted the manual ways of detecting the disease. The hybrid DL systems demonstrate the potential to operate with great volumes of data and to obtain automatic features to achieve better accuracy in the classification of plant diseases. The research in the areas of ML and DL is discussed in this section in order to illustrate their methods and performance in plant disease recognition (Tripathi et al. 2023) summarized in Table 1.

**TABLE 1:** Limitations of the related work.

References	Year	Image‐based or feature‐based	Number of images	Augmentation	Method	Results (%)	Limitation
Mostafa et al. (2021)	2021	Image‐based	2889	Yes	ResNet‐101	97.74	(1) Accuracy can be improved (2) NPV, FPR, and FNR not calculated (3) XAI not applied
Al Haque et al. (2019)	2019	Image‐based	10,000	Yes	CNN	95.61	(1) Accuracy can be improved (2) Specificity, NPV, FPR, and FNR not calculated (3) XAI not applied
Jain et al. (2023)	2023	Image‐based	6000	No	CNN	95.90	(1) Accuracy can be improved (2) Specificity, NPV, FPR, and FNR (3) No augmentation (4) XAI not applied
Thangaraj et al. (2023)	2023	Image‐based	2300	No	DenseNet169	96.12	(1) Accuracy can be improved (2) Small Dataset (3) Specificity, NPV, FPR, and FNR not calculated (4) No augmentation (5) XAI not applied
					DenseNet121	89.89	
					InceptionV3	89.46	
					Xception	90.54	
World Scientific and Engineering Academy and Society (WSEAS) (2004)	2023	Image‐based	1834	Yes	EfficinetNet‐B3	94.93	(1) Accuracy can be improved (2) Small dataset (3) Specificity, NPV, FPR, and FNR not calculated (3) XAI not applied
					VGG‐16	90.10	
					Res‐Net50	92.10	
					InceptionV3	93.70	
Kilci and Koklu (2024)	2024	Image‐based	3784	Yes	SqueezeNet	95.6	(1) Accuracy can be improved (2) Specificity, NPV, FPR, and FNR not calculated (3) XAI not applied

ML technology has an enormous role in the development of plant disease detection systems. Previous studies have extracted features of plant images and have used Support Vector Machines (SVM) and combined with K‐Nearest Neighbors (KNN) and random forest algorithms to classify the images. The collaboration of the predictive analytics and the ML algorithms used by Merida and Palmateer (2006) resulted in the system of guava tree disease monitoring. The application of image processing, which was accomplished by extracting the texture and the process of obtaining the shape and color features, produced appropriate outputs. The utility of classical ML methods is restricted by extraction challenges in the process of features. These methods together lead to high computational cost as well as high failure to detect most of the time, especially in the process of handling complex datasets. A team of researchers has explored combined analysis techniques that combine ML and image enhancement processes to enhance predictive accuracy. These methods are still used to solve scalability problems and intensive processing needs.

The revolution in plant disease detection process has come with the introduction of DL, which is able to extract features automatically. The CNN and DL have the advantage of being able to extract more complex features directly through raw image data, allowing them to be better when handling large and multifaceted data. An article in the Applied Sciences (Mostafa et al. 2021) presented excellent results that they managed to get 97.74% accuracy using their designed CNN model to diagnose diseases in guava plants. According to this model, DL was found to be able to effectively handle the complex and diverse patterns of the data associated with guava diseases and, therefore, exhibits the powerful potential of enhancing the process of disease detection.

The use of TL methods involving networks such as ResNet and VGG‐16 and CNN has provided the best accuracy rates with less data. The study of WSEAS (2004) depicted that the use of TL in the diagnosis of cucumber disease presented 94.93% accuracy outputs. By using the TL methods, the computers produce credible responses and with less demand for the large quantity of computational power, the computer works at small data sets.

DL approaches, especially those incorporating CNN with TL ones, have already led to significant advancement in the technology of plant diseases detection. Computational efficiency of ML methods which employ these techniques is high, in addition to high accuracy rates, in comparison with the older traditional methods of ML. These methods have a bright future in the agriculture automatization, as they can be observed in an ongoing development of disease monitoring and management methods.

To find pomegranate fruit disease, the ROI in Deshpande et al. (2014) is improved, resized, and isolated to pull shadows from the background. K‐means is then used to cluster the data. Applying fundamental color transformation techniques in picture processing helped to identify the affected leaf areas, therefore enabling guava disease detection. The classification in the study in Thilagavathi and Abirami (2017) was done using SVM and the KNN approach. Apple diseases were found using the spot segmentation approach. Feature fusion and extraction were also part of this approach. The study (Khan et al. 2019) used the decorrelation technique to combine the extracted characteristics. The author of Gasanov et al. (2020) also employed a soil‐based study to help identify which soil indicator is necessary for generating plant yield. Much like in the prior example, historical weather forecasting could be a useful tool for plant monitoring systems to avoid natural disasters. The good results DL research regularly generates make it very sought after. Big data simplifies the training of DL models to produce forecasts for automated detection. A related study used over 54,000 images of 14 different crop diseases, representing 26 different disease categories. The proposed deep convolutional neural network (DCNN) attained 99.35% accuracy on the specified test dataset. Finally, in Mohanty et al. (2016), a smartphone app for the automatic identification of crop diseases was shown. A similar large data set was employed to identify plant diseases. The open dataset comprised 25 distinct plant categories, spanning over 87,000 images. Among the various DCNN designs, the best‐performing network achieved an accuracy of 99.53%. The findings indicate that this tool model can detect plant diseases in real time (Ferentinos 2018). The researchers suggested a visualization method and their CNN to address the symptom‐based deficiencies of earlier architectures. These discrepancies were found by researchers.

Results were better in terms of (Saleem et al. 2019) when plant diseases were identified using altered networks. The models employing TL and complex features from current architectures have basic structures. The outcomes of the transfer‐learning approach were worse than those of the deep‐feature‐based classification employing SVMs and other ML classifiers.

Among them are AlexNet, VGG‐16, and VGG‐19. Although recorded in real time, some diseases and symptoms were not included in the publicly accessible databases. We employed data augmentation to circumvent this limitation. This required combining a single input image of a leaf with multiple viewpoints, each reflecting a different condition associated with the same leaf. It also covered a large spectrum of diseases that can damage leaves. Augmentation‐derived forecasts increased accuracy by 12%. Furthermore, in Barbedo (2019), the Plant‐Village dataset had many annotated photos of apple black rot; thus, data augmentation was proposed as a remedy for the data constraints. With a 94% accuracy (Wang et al. 2017), the VGG‐16 model surpassed all other trained DL models.

### 2.1 Research Gap

- Lower accuracy: Previous studies (Mostafa et al. 2021) report lower classification accuracy than our proposed model, showing the need for more precise guava disease classification approaches.
- Limited dataset size: Several referenced studies (Mostafa et al. 2021; WSEAS 2004) used small datasets, which may affect model generalizability.
- Incomplete performance metrics: Key performance metrics, such as misclassification rate, specificity, NPV, FPR, and FNR, have not been reported in previous works (Mostafa et al. 2021; Kilci and Koklu 2024; WSEAS 2004; Al Haque et al. 2019), which limits a comprehensive evaluation of their models
- Lacks transparency and fairness: Other related works (Mostafa et al. 2021; Kilci and Koklu 2024; WSEAS 2004; Al Haque et al. 2019; Jain et al. 2023; Thangaraj et al. 2023) are able to classify the disease but fail to use XAI techniques making them lack transparency and fairness. This may be explained by the absence of interpretability, which makes the model decisions opaque and, therefore, a clear difference in the approaches to achieving both the accuracy, as well as the explainability of reasoning.

### 2.2 Contribution of Proposed Model

- The learning transfer model development: A DL model ResNet‐101 is a state‐of‐the‐art CNN that has been improved with a TL implementation. The approach allows the model to use pre‐trained weights, and it is modified to identify guava diseases and minimizes the training time and enhances performance.
- Guava disease detection system: The system recognizes and tabulates three kinds of guava diseases: Anthracnose, fruit fly, and healthy Guava. In the case of farmers operating automatic detection tools, the system will give them a proper monitoring system where they can monitor the health of their crop and make a timely decision to prevent losses.
- Data augmentation of a balanced dataset: A fully implemented data augmentation scheme was undertaken to strengthen the data and overcome the first imbalance of classes. 4632 was the result of the methods of rotation, flipping, and slight changes of the brightness and led to the even distributions of the amounts of the samples of Anthracnose, Fruit Fly, and Healthy guava. This more diversified and balanced data contributed more visual patterns to the model to enable it to generalize and further minimize the chances of overfitting, and eventually enhancing the classification accuracy and reliability.
- High accuracy: The applied ResNet‐101 model has a very high accuracy of 98.48% that is better than all the models presented in the literature (Mostafa et al. 2021; Kilci and Koklu 2024; WSEAS 2004; Jain et al. 2023). Our methodology has a success in diagnosis of a guava disease as the accuracy was 98.48.
- Comprehensive evaluation using performance metrics: In addition to accuracy, the model evaluates multiple performance metrics, including misclassification rate, specificity, NPV, FPR, and FNR. An assessment based on these metrics provides comprehensive knowledge about the real‐world dependability and effectiveness of the designed model.
- Explainable AI with Grad‐CAM: Grad‐CAM is used in the model to simplify the predictions made by the DL. This XAI technique creates heatmaps that visibly indicate the most important regions of the image for the decision that the model will make. Grad‐CAM makes predictions transparent and identifies points of infection or pest damage that motivate individual predictions, which in turn enhance the level of trust in the model for real‐life settings, thereby vindicating the effectiveness of the model.

## 3 Proposed Methodology

**FIGURE 2:**
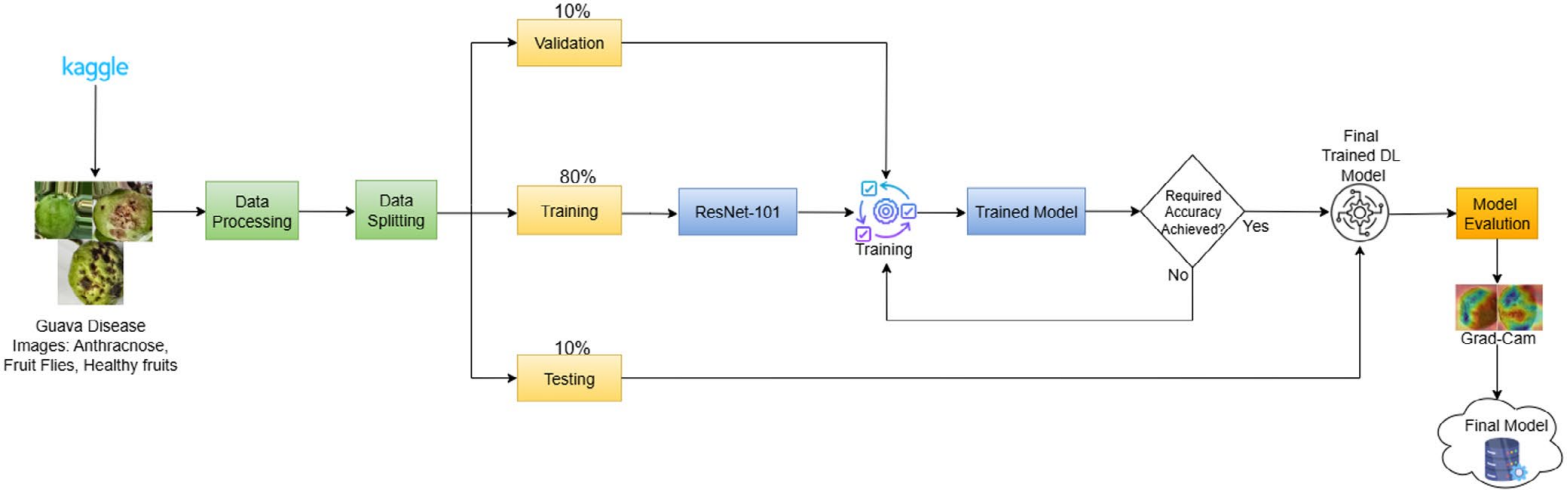
Architecture of the proposed model.

Figure 2 illustrates the whole architecture of the proposed guava disease detection system. The processing of Guava fruit images starts with the obtaining of the data on the Guava fruit at the Kaggle dataset (Jain et al. 2023), which includes three classes of images, namely, Anthracnose, Fruit Fly, and Healthy Guava samples. The images are then manipulated to satisfy the requirements of the DL system through preprocessing, including the resizing to 224 × 224 pixels, turning them into tensors, and normalizing them. The processed data is then divided into three components, namely 80% is used in the training, 10% in the validation, and 10% in the testing with all of the classes evenly represented. The ResNet‐101 network is pre‐trained on ImageNet and then fine‐tuned on training data in training and a hyperparameter choice influenced by validation data to serve as a means to prevent overfitting.

The model may fail to provide the intended accuracy, and thus the training loop reiterates until one gets the desired accuracy. After successful training of the model, generalization is properly tested on the hidden testing set. Grad‐CAM is added as well to improve interpretability, which generates heatmaps to describe which features of the image are the most influential on every prediction. This is so that the network is listening to the symptomatic aspects of the ailment and not the background. The developed end‐to‐end architecture will develop a robust, interpretable DL model capable of classifying images of the guava fruits and assigning them to their respective health labels successfully and can be utilized in the real‐world scenario in agricultural disease detection.

### 3.1 Dataset

This original data sample of guava disease was 3784 images categorized in three groups namely the Anthracnose (1544 images), Fruit Fly (1312 images), and the Healthy Guava (928 images). One certain method of data augmentation was used to correct the imbalance and improve the generalization of the model with the addition of other data types, by rotation, flipping, or changing the brightness. This was done to achieve a perfect number of images per class (1544 images per class), resulting in a total of 4632 images. Table 2 describes that the augmented dataset was then split into 80% training (3708 images), 10% validation (462 images), and 10% testing (462 images), with each set equally represented by all the classes. This enriched and balanced dataset provided a sufficient basis for training and testing the proposed DL model, resulting in increased stability and robustness.

**TABLE 2:** Dataset parameters.

Classes	Train (80%)	Validation (10%)	Test (10%)	Total images
Anthracnose	1236	154	154	1544
Fruit Fly	1236	154	154	1544
Healthy guava	1236	154	154	1544
Grand total	3708	462	462	4632

**FIGURE 3:**
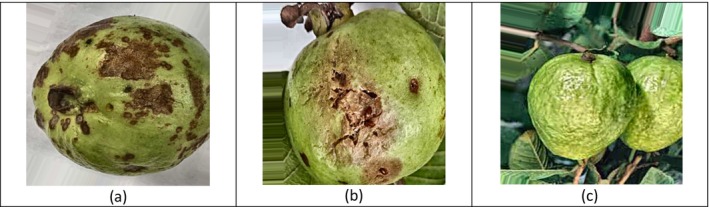
Sample images of (a) Anthracnose, (b) fruit fly, and (c) healthy guava.

The sample pictures of all three guava types that were used in the study are depicted in Figure 3. In Image (a), there is a guava with Anthracnose infection with dark sunken spots on the surface of the fruit that are prominent. Image (b) shows a ripe guava that is destroyed by the Fruit Fly, with some puncture holes and internal decay. Image (c) is a healthy guava with smooth and consistent skin and no spots in sight. These samples exemplify the visual clues of every class and represent the diversity present in the data being used, which enables the DL model to distinguish between diseased and healthy fruit quite well.

### 3.2 Transfer Learning

In TL, data preprocessing is essential to ensure that input images are properly formatted for the pre‐trained model. The model is trained to classify the images into the three defined categories. If the model meets the predefined performance criteria, it is finalized and stored for future deployment. If the performance criteria are not met, the model undergoes retraining to improve its accuracy. Once validated, the trained model is deployed as a reliable solution for guava disease detection, ensuring efficient and accurate classification.

#### 3.2.1 ResNet‐101

**FIGURE 4:**
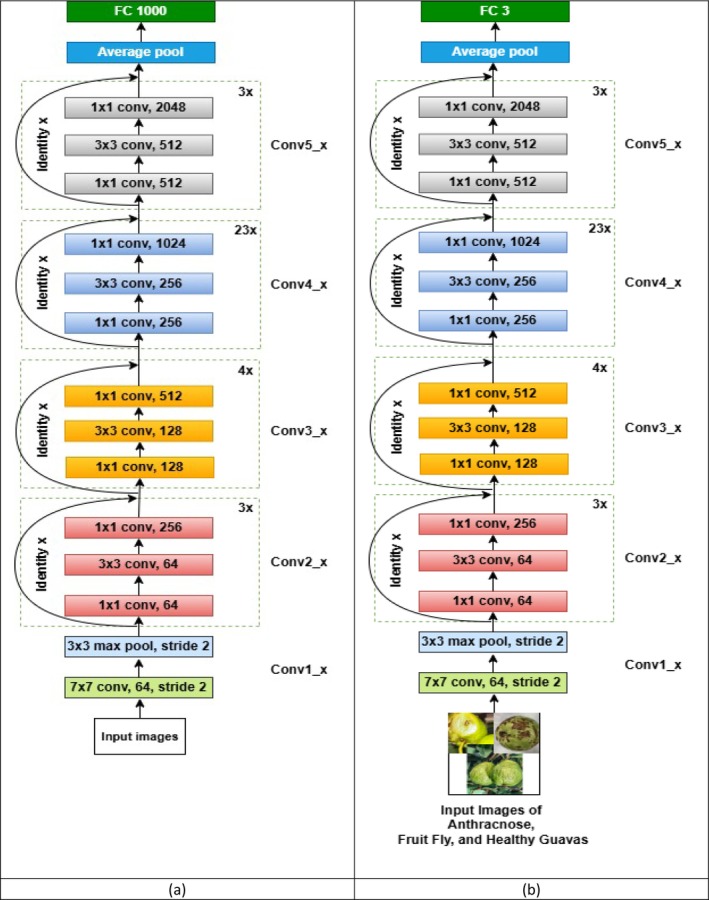
(a) ResNet‐101 architecture for the proposed model, (b) modified ResNet‐101 architecture for the proposed model.

ResNet‐101 is an advanced version of the original ResNet‐101 architecture. Initially designed for large‐scale image classification tasks with 1000 categories, the architecture of ResNet‐101 is shown in Figure 4. This deep architecture employs residual connections, which help mitigate the vanishing gradient problem, allowing the model to learn complex patterns effectively, even in very deep networks. Figure 4a illustrates the ResNet‐101 architecture for the proposed model, and Figure 4b shows the modified ResNet‐101 architecture for the proposed model.

The ResNet‐101 model has been modified to classify three specific types of guava diseases: Anthracnose, Fruit Fly, and Healthy guava. This adaptation of the original model, tailored for classifying only three classes, is illustrated in Figure 4b. By fine‐tuning the ResNet‐101 architecture, we focus on detecting the distinct features of each disease, enabling the model to make accurate predictions on guava fruit conditions.

The ResNet‐101 model requires input images sized 224 × 224 × 3, where each image is resized to 224 pixels by 224 pixels, with three color channels (RGB). The architecture is composed of multiple residual blocks, each containing several layers, which enable the model to learn hierarchical features essential for distinguishing between different disease states.

## 4 Simulation and Results

The training of the model was done in Google Colab with the help of acceleration with a GPU (Tesla T4). Python 3.11.12, PyTorch 2.6, torchvision 0.21, NumPy 2.0.2, Matplotlib 3.10, PIL 11.1.0, and CUDA 12.4 were all part of the software setup.

The hyperparameters that will be employed in training in the proposed model, which will employ a modified ResNet101, are summarized in Table 3. This simulation was trained with the mini‐batch of 16, the optimal number of epochs of 5, the learning rate of 0.0003, and Adam optimization algorithm. The mini‐batch size implies that the model will be computing 16 samples at a time in an attempt to generate gradients and update its parameters. It was observed in training the model under different epochs that the best results were obtained with 5 epochs, which is considered as a complete run through the entire training material. Adam optimizer, which is known to be efficient and able to handle noisy data, was used with the learning rate of 0.0003 to stabilize and effectively train the data.

**TABLE 3:** Hyperparameters of the proposed model.

Hyperparameters	Value
Number of epochs	5
Learning rate	0.0003
Optimizer	Adam
Size of input images	224 × 224 pixels
Batch size	16

**FIGURE 5:**
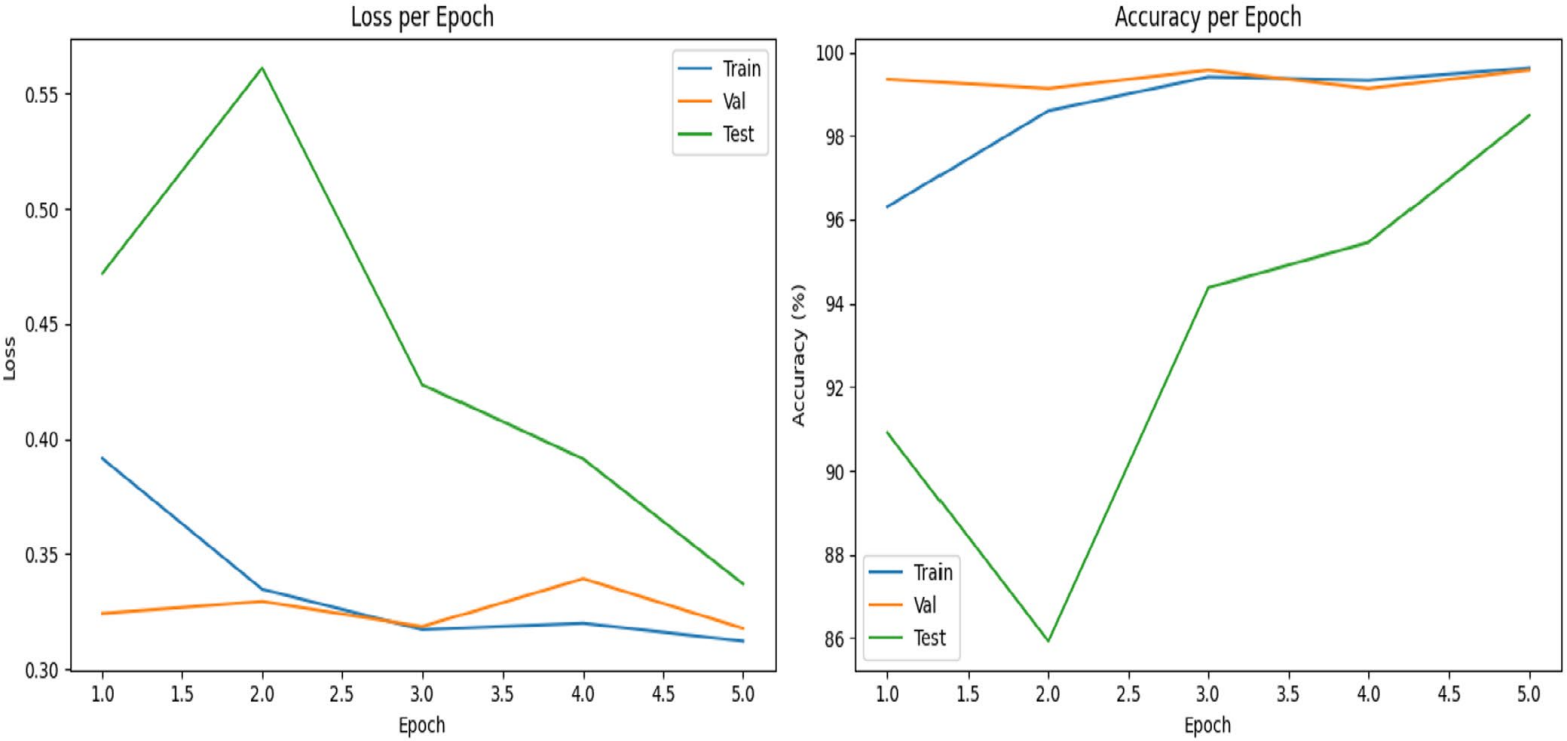
Training and testing loss and accuracy.

Figure 5 shows the training performance of the proposed guava disease detection model in five epochs, showing both the loss and accuracy variations. The left curve is the loss curve, which is used to show how the errors of prediction are varying during the training process. The training loss also reduced but at a slow pace and was approximately 0.31, the validation loss was low and constant at around 0.32–0.34, this means that the loss was working and the learning did not overfit. The test loss was initially higher at an approximation of 0.48, and it momentarily increased to a new high of 0.56 in the second epoch after which it went back to a level of about 0.34 which is an improvement in generalization as compared to the unseen images. The accuracy curve is used to complement these results indicating that training and validation accuracy had risen to more than 96% and nearly 99%, respectively. The test accuracy was initially during the first epoch of approximately 91%, it declined to 86% during the second epoch and it increased drastically to more than 98% during the final epoch. The combination of these graphs has established that the ResNet‐101 model had good predictive capacity with low error and high accuracy on images of guava.

### 4.1 Accuracy

It measures the overall correctness of a classification model by calculating the ratio of correctly predicted instances (true positives and true negatives) to the total instances. It is expressed as:

(1) $$\text{Accuracy}=\frac{\mathrm{ṬP}+ṬN}{\mathrm{ṬP}+FP+\mathrm{FN}+ṬN}\times 100$$

A higher accuracy indicates better model performance, but it may not be reliable for imbalanced datasets, where additional metrics like precision and recall are necessary for a comprehensive evaluation.

### 4.2 Misclassification Rate

It provides an evaluation metric by determining the percentage of instances receiving false classification. It is mathematically defined as:

(2) $$\text{Misclassification rate}=\frac{FP+\mathrm{FN}}{\mathrm{ṬP}+FP+\mathrm{FN}+\mathrm{TN}}\times 100$$

The measurement of accuracy reflects correctly classified instances that can also be expressed through the following formula:

Misclassification rate=1−Accuracy

The MCR drops when a model performs better because it recognizes a lower number of cases incorrectly. The misclassification rate offers specific evaluation capabilities for classification errors when using it in combination with accuracy and additional performance metrics.

### 4.3 Specificity

It measures the model's ability to correctly identify negative cases. It is calculated as:

(3) $$\text{Specificity}=\frac{\mathrm{ṬN}}{\mathrm{ṬN}+FP}\times 100$$

### 4.4 Recall

It measures the model's ability to correctly identify positive cases. It is calculated as follows:

(4) $$\text{Recall}=\frac{\mathrm{ṬP}}{\mathrm{ṬP}+\mathrm{FN}}\times 100$$

### 4.5 Precision

It measures the proportion of correctly predicted positive cases among all predicted positives. It is calculated as follows:

(5) $$\text{Precision}=\frac{\mathrm{ṬP}}{\mathrm{ṬP}+FP}\times 100$$

### 4.6 Negative Predictive Value

It measures the proportion of correctly predicted negative cases among all predicted negatives. It is calculated as follows:

(6) $$\text{Negative prediction value}=\frac{\mathrm{ṬN}}{\mathrm{ṬN}+\mathrm{FN}}\times 100$$

### 4.7 False Positive Rate

It is also known as the false positive ratio, measures the proportion of negative instances that were incorrectly classified as positive. It is calculated as:

(7) $$\text{False positive rate}=\frac{FP}{FP+ṬN}\times 100$$

### 4.8 False Negative Rate

It measures the proportion of actual positive cases that the model incorrectly classifies as negative. It is calculated as follows:

(8) $$\text{False negative rate}=\frac{\mathrm{FN}}{\mathrm{ṬP}+\mathrm{FN}}\times 100$$

### 4.9 F1 Score

It is the harmonic mean of precision and recall (sensitivity), balancing both metrics to provide a single performance measure, especially useful when the dataset is imbalanced. It is calculated as:

(9) $$F1-\text{Score}=2\times \frac{\text{Precision}\times \text{Sensitivity}\;}{\text{Precision}+\text{Sensitivity}}$$

The performance of the model is evaluated through several well‐established statistical metrics, as outlined in Equations ((1), (2), [Link], (3), (4), (5), (6), (7), (8), (9)) to assess its classification accuracy (Murtaza et al. 2020; Tharwat 2021).

**FIGURE 6:**
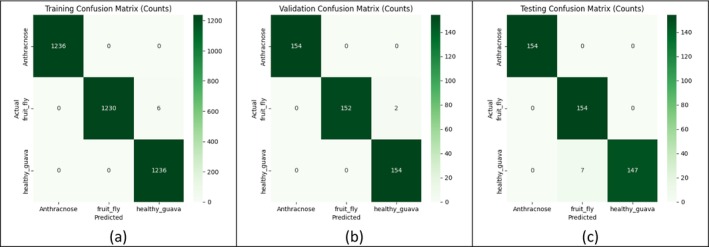
Performance evaluation of the proposed model through confusion matrices for (a) training, (b) validation, and (c) testing dataset.

Figure 6 illustrates the degree to which the model identified each guava category during training, validation, and testing. During the training phase, the model nearly exactly recognized each image. Anthracnose had 1236 correct detections and no wrong guesses, and healthy guava had 1236 correct detections and six wrong guesses. The fruit fly had 1230 correct detections and six incorrect guesses. In the case of the validation set, the accuracy remained very high. All 154 cases were correctly identified as anthracnose, with only two false positives. Fruit fly and healthy guava also had 154 correct with two false images. The model again demonstrated good performance on the testing set, which consisted entirely of new images. Anthracnose hit 154 correct and 0 false, fruit fly hit 154 correct, and seven additional false positives, and healthy guava hit 147 correct and seven missed hits. In general, these confusion matrices demonstrate that even with end testing, the model maintains its high accuracy during training, and it can be depended upon to differentiate between healthy fruit and those that are diseased with Anthracnose or Fruit Fly damage.

Table 4 provides a closer examination of the proposed model, ResNet‐101, in terms of training, validation, and testing performance. The model used during training achieved a high accuracy of 99.84% with a minor misclassification rate of 0.16%. Recall, as well as precision, scored similarly, and the F1 score of 0.9984 represents a great balance between recall and precision. The results of the validation phase are also quite impressive, with 99.57% accuracy and an extremely low misclassification rate of 0.43%. Specificity and NPV were both above 99%, indicating that the model was reliable in differentiating between diseased and healthy guavas. The model also performed well on the unseen testing data, achieving 98.48% accuracy and an F1 score of 0.9848. Specificity remained high at 99.24%, and the false‐positive and false‐negative rates were 0.76% and 1.52%, respectively. These findings indicate that the trained model not only learns efficiently with the training set but also generalizes to new images, providing the same, accurate disease detection that can be applied to practice in the agricultural industry.

**TABLE 4:** Proposed model performance evaluation.

Evaluation matrices	Training	Validation	Testing
Accuracy	99.84%	99.57%	98.48%
Misclassification rate	0.16%	0.43%	1.52%
Specificity (selectivity)	99.92%	99.78%	99.24%
Recall (sensitivity)	99.84%	99.57%	98.48%
Precision (PPV)	99.84%	99.57%	98.55%
NPV	99.92%	99.78%	99.26%
FPR	0.08%	0.22%	0.76%
FNR	0.16%	0.43%	1.52%
F1 score	0.9984	0.9957	0.9848

**FIGURE 7:**
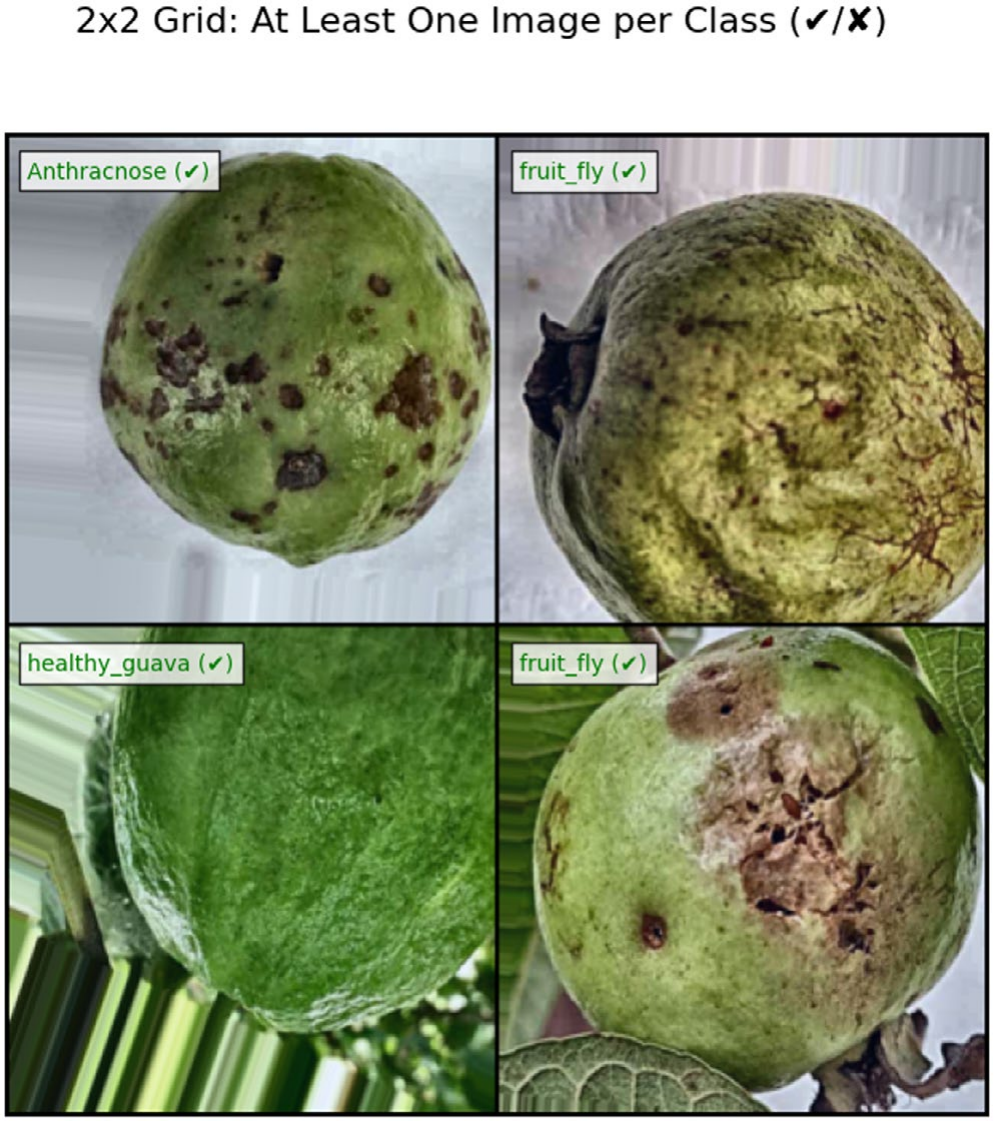
2 × 2 grid of guava images: Anthracnose, fruit fly, and healthy guava (labeled with tick for classification).

Figure 7 highlights the model's classification accuracy using sample images from the dataset. The model will remain in use as long as it satisfies its performance standards; however, it will undergo additional training to fulfill performance requirements if it does not meet them. The model proceeds to analyze the testing dataset after successful training completion. The learned model becomes accessible on the cloud, where it will be available for future use.

**FIGURE 8:**
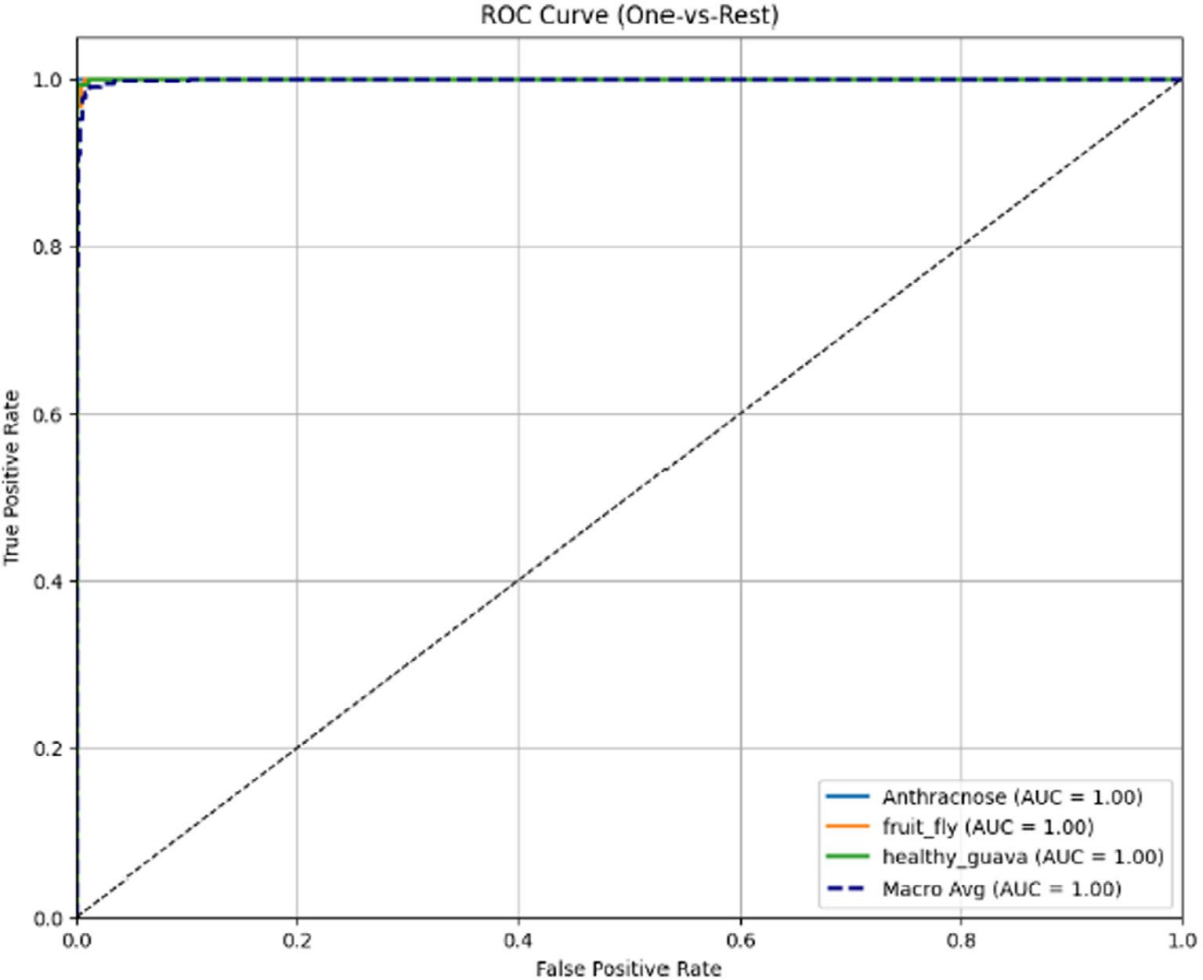
ROC curves of the proposed model.

The receiver operating characteristic (ROC) analysis of the three guava types, Anthracnose, Fruit Fly and Healthy Guava, was conducted using a one‐versus‐rest strategy (Figure 8). The ROC curve is used to measure how well a classifier performs at all decision thresholds by graphing the Recall against the FPR (1 − Specificity) on a graph. In Figure 8, the curves of all three classes are tightly grouped around the top‐left corner, with the true positive rate clustering closely with the FPR rate, which is close to 1.0 and 0.0, respectively. This type of pattern indicates that the model consistently identifies diseased and healthy fruit with the fewest false alarms.

The area under the curve (AUC) of both classes and the macroaverage is 1.00, indicating approximately perfect separability of positive and negative samples of all categories. This remarkable finding confirms that, even with varied classification thresholds, the proposed ResNet‐101 model can significantly classify Anthracnose, Fruit Fly damage, and healthy guavas correctly. The overlapping of the curves also reflects balanced performance, whereby no class is affected at the expense of the other. This result confirms the strength of the trained network and points to the accuracy of the trained network in an application to real‐life guava disease recognition and timely intervention in the agricultural sector.

**FIGURE 9:**
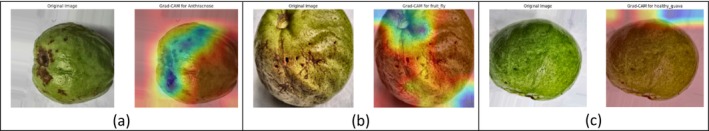
Grad‐CAM visualizations of the proposed model highlighting diseased regions of guava: (a) Anthracnose, (b) Fruit Fly, (c) Healthy Guava.

The Grad‐CAM visualizations in Figure 9 demonstrate how the proposed model identifies the key components of the guava images during classification. The areas that have been highlighted in (a) refer to the lesions characteristic of Anthracnose, and those in (b) are those that have been infested by Fruit Fly. In (c), a picture is used to depict a Healthy Guava, and there are no important areas of disease shown. These heatmaps confirm that when predicting the locations in the image where the disease is actually present, the network is indeed focusing on those areas, providing a clear visual indication of the model's validity and interpretability.

Table 5 compares the proposed ResNet‐101 model with previous studies on guava disease detection. Past work achieved accuracy between 90% and just under 98%. For example, the 2021 ResNet‐101 in Mostafa et al. (2021) achieved 97.74%, while CNN‐based models in Al Haque et al. (2019), Jain et al. (2023), and Thangaraj et al. (2023) scored 94.93%–96.00%, and EfficientNet‐B3 in WSEAS (2004) reached 95.00%. Ensemble methods, such as SqueezeNet with Gradient Boosting (Kilci and Koklu 2024), achieved up to 95.6%, remaining competitive with top single‐network solutions.

**TABLE 5:** Comparative analysis of existing DL models with proposed model.

References	Year	Method	Accuracy (%)	Misclass‐ification rate (%)	Specificity (selectivity %)	Recall (sensitivity %)	Precision (PPV %)	NPV %	FPR %	FNR %	F1 score
Mostafa et al. (2021)	2021	ResNet‐101	97.74	2.26%	0.9964	—	0.9884	—	—	—	0.9883
Al Haque et al. (2019)	2019	CNN	95.61	4.39%	—	95.40%	94.94%	—	—	—	97.49%
Jain et al. (2023)	2023	CNN	95.90	4.10%	—	Healthy = 95.88% LeafSpot = 95.53% GuavaRust = 96.14% GuavaCanker = 96.04%	Healthy = 96.06% LeafSpot = 94.57% GuavaRust = 94.20% GuavaCanker = 98.04%	—	—	—	Healthy = 95.49% LeafSpot = 94.49% GuavaRust = 94.98% GuavaCanker = 96.97%
Thangaraj et al. (2023)	2023	CNN	96.12	3.88%	94%	—	96%	—	—	—	0.94
WSEAS (2004)	2023	EfficinetNet‐B3	94.93	5.07%	—	—	—	—	—	—	—
Kilci and Koklu (2024)	2024	SqueezeNet	AdaBoost = 82.7% Grandient Boosting = 95.6%	—	—	—	—	—	—	—	AdaBoost = 0.827 Grandient Boosting = 0.956
Proposed model	2025	ResNet‐101 With Grad‐CAM	98.48%	1.52%	99.24%	98.48%	98.55%	99.26%	0.76%	1.52%	0.9848

These findings form the basis of the proposed model that includes data augmentation to equalize the classes and XAI with the help of Grad‐CAM visualization. The accuracy of the method is high (98.48), the misclassification rate is low (1.52), and the supporting values have very good values such as 99.24% specificity, 98.55% precision, 99.26% NPV, and F1 score is 0.9848 and is hence better than all the above methods in practically all the relevant metrics. These findings indicate the efficiency of the balanced data augmentation scheme when combined with the powerful ResNet‐101 backbone, and clear model interpretation could be presented as a more legitimate and comprehensible answer to the real‐life agricultural application of guava disease detection.

## 5 Discussion

The findings of the current paper prove that the ResNet‐101 model with the help of specific data augmentation and the use of the fine‐tuning technique can be successfully applied and used to differentiate between Anthracnose, Fruit Fly damage, and healthy guavas. The results of the model in terms of training, validation, and unknown test data are indicative that the chosen TL strategy forms an excellent basis for the real application of guava disease detection. Nevertheless, there are still some crucial factors that cannot be overlooked that can be taken into account to enhance the soundness, justice, and practicality of the system.

One of the main flaws of the current paper is that it uses a single publicly available dataset, which was taken under controlled imaging conditions. Although these are enough to allow preliminary validation, they cannot capture the same level of variation as experienced in the real agricultural setting, where the lighting, background conditions, orientation of fruits, and overall camera quality can vary significantly. Further research that included images collected in the field in various geographical locations would bring more visual diversity and would aid in the assumption that the model can be generalized in practice in typical farming conditions. This will be a step beyond the bounds of the current study, but will be a crucial move towards creating a deployable, farmer‐centric solution.

Strategies of model validation can also be extended. The existing 80/10/10 train‐validation‐test division is an agreed‐upon method of TL research and offers sound evidence of generalization. However, less general methods like k‐fold cross‐validation or cross‐dataset evaluation might provide further information on model stability. These approaches are computationally intensive to deep networks like the ResNet‐101 and could not be done in this revision cycle, but as directions to methodological improvement in the future.

The convergence of the training behavior in this experiment depends on the fact that it is stable with minimal differences in the training and validation losses. This can be interpreted as the hyperparameter settings and regularization options working effectively to a great extent to prevent overfitting. In order to enhance the model's robustness, methods like early stopping, dropout, or extra weight‐decay regularization could be incorporated in future work. The nature‐inspired optimization methods are also seen as promising in enhancing the tuning of DL models according to the recent literature. Such approaches as Greylag Goose Optimization, Adaptive Dynamic Optimization, and Comment Feedback Optimization have been effectively used to improve the convergence stability and search performance in complex prediction tasks (El‐Kenawy et al. 2025; Alhussan et al. 2025; El‐Kenawy, Alhussan, et al. 2024; El‐Kenawy, Khodadadi, et al. 2024). Adding the same strategy to the model in the future can further enhance generalization, especially in case of training on a larger or more heterogeneous set of data.

There are ethical and practical issues that need to be tackled in addition to methodology issues when implementing AI‐based disease detection in agricultural environments. With differences in the data spread throughout regions, it is possible that unintentional biases will be introduced, and issues concerning the ownership of data and fairness will become more critical as these systems become more widespread. Despite a certain degree of insight ability provided by Grad‐CAM, where image sections that shape predictions are identified, deep neural networks are more or less enigmatic, and users might need extra explainability to agree with the choices made by the models.

Another issue of concern is deployment feasibility. Although ResNet‐101 has a high accuracy, it is a deep network with high computational cost and hence it might not be applicable to mobile or edge devices that are common with farmers in low resource settings. Richer lightweight architectures, pruning, quantization, or model‐compression methods might be required to realize real‐time inference on resource‐constrained hardware. Subsequent research ought to involve measuring the inference time, memory footprint, and energy expenditure to establish the feasibility of the implementation of such a model in an actual farming setting.

Last, additional gains can be made through investigating the use of multimodes imaging processes such as hyperspectral imaging, thermal imaging, and near infrared imaging, which can be used to determine early disease signs that cannot be observed with conventional RGB images. The domain adaptation and semi‐supervised learning techniques are also promising to be able to make the model capable of adapting to new conditions of the field with the minimum required supplementary labeled data. These extensions would help in providing a more flexible and scalable system that can address the needs of the diverse farming communities.

## 6 Conclusion and Future Works

The present research suggested a DL model to estimate guava disease with a great degree of accuracy with the assistance of a ResNet‐101‐based model. Specific data augmentation was used to increase the number of samples of the initial dataset of 3784 images up to 4632 images, as well as to provide a more equal representation of each category, which strengthened the model. The findings on the improved data, following training, validation, and testing, were good; the model had training, validation, and testing accuracy of 99.84, 99.57, and 98.48, respectively. Grad‐CAM XAI was further incorporated to make further application more practical, allowing visual highlighting of the diseased part of the fruit and making decisions at a glance about the presence of disease in fruit. These combined techniques point to the fact that the proposed system will be capable of reliably identifying healthy guavas and infected ones with Anthracnose or Fruit Fly, which can be scaled and applied in a real‐life agricultural setting by the farmers as a tool of detecting diseases in a new crop early enough.

The future research will build on the present study by introducing federated learning (FL) into the framework so that a combination of farms or research centers can collaboratively train and, notwithstanding that, there is no necessity in sharing raw image data. This privacy protecting approach will help the model to be adjustable to different environmental factors and regional distinctions without compromising the data security. Another approach that will be contemplated to offer a more understanding and detailed visual representation as compared to GradCAM is more sophisticated XAI approaches. Potential approaches are Layer‐wise Relevance Propagation (LRP), Shapley Additive Explanations (SHAP), and Local Interpretable Model‐Agnostic Explanations (LIME), each with their own strengths in being able to identify how deep networks make their choices. The combination of these XAI techniques with FL will raise the rates of transparency, interpretability, and trust, will enable the identification of diseases in different crops and geographic locations on a scale, in real‐time, and will enable more sustainable agricultural management.

## Author Contributions

**Muhammad Ahmed:** validation (equal), visualization (equal). **Fahad Ahmed:** supervision (equal), validation (equal), visualization (equal). **Naila Sammar Naz:** investigation (equal), methodology (equal), writing – review and editing (equal). **Tehseen Mazhar:** editing, formating and writing. **Muhammad Adnan Khan:** resources (equal), validation (equal), visualization (equal). **Muhammad Amir Khan:** data curation (equal), investigation (equal), validation (equal), visualization (equal). **Amel Ksibi:** resources (equal), supervision (equal). **Mohamed Abbas:** software (equal), validation (equal), visualization (equal).

## Funding

This study was funded by Princess Nourah bint Abdulrahman University Researchers Supporting Project number (PNURSP2025R759), Princess Nourah bint Abdulrahman University, Riyadh, Saudi Arabia.

## Conflicts of Interest

The authors declare no conflicts of interest.

## Data Availability

The data used to support the findings of this study are available from the corresponding authors upon request.

## References

[fsn371348-bib-0001] Al Haque, A. F. , R. Hafiz , M. A. Hakim , and G. R. Islam . 2019. “A Computer Vision System for Guava Disease Detection and Recommend Curative Solution Using Deep Learning Approach.” In 2019 22nd International Conference on Computer and Information Technology (ICCIT). IEEE.

[fsn371348-bib-0002] Alhussan, A. A. , E. S. M. El‐Kenawy , D. S. Khafaga , A. H. Alharbi , and M. M. Eid . 2025. “Groundwater Resource Prediction and Management Using Comment Feedback Optimization Algorithm for Deep Learning.” IEEE Access 13: 169554–169593.

[fsn371348-bib-0003] Almadhor, A. , H. Rauf , M. Lali , R. Damaševičius , B. Alouffi , and A. Alharbi . 2021. “AI‐Driven Framework for Recognition of Guava Plant Diseases Through Machine Learning From DSLR Camera Sensor Based High Resolution Imagery.” Sensors 21, no. 11: 3830. 34205885 10.3390/s21113830 PMC8198251

[fsn371348-bib-0004] Barbedo, J. G. A. 2019. “Plant Disease Identification From Individual Lesions and Spots Using Deep Learning.” Biosystems Engineering 180: 96–107.

[fsn371348-bib-0005] Deshpande, T. , S. Sengupta , and K. Raghuvanshi . 2014. “Gr Ading & Identification of Disease in Pomegranate Leaf and Fruit.” International Journal of Computer Science and Information Technologies 5, no. 3: 4638–4645.

[fsn371348-bib-0006] El‐Kenawy, E. S. M. , A. A. Alhussan , M. M. Eid , and A. Ibrahim . 2024. “Rainfall Classification and Forecasting Based on a Novel Voting Adaptive Dynamic Optimization Algorithm.” Frontiers in Environmental Science 12: 1417664.

[fsn371348-bib-0007] El‐Kenawy, E. S. M. , A. Ibrahim , A. A. Alhussan , D. S. Khafaga , A. E. M. Ahmed , and M. M. Eid . 2025. “Smart City Electricity Load Forecasting Using Greylag Goose Optimization‐Enhanced Time Series Analysis.” Arabian Journal for Science and Engineering 25: 1–19.

[fsn371348-bib-0008] El‐Kenawy, E. S. M. , N. Khodadadi , S. Mirjalili , A. A. Abdelhamid , M. M. Eid , and A. Ibrahim . 2024. “Greylag Goose Optimization: Nature‐Inspired Optimization Algorithm.” Expert Systems With Applications 238: 122147.

[fsn371348-bib-0009] Ferentinos, K. P. 2018. “Deep Learning Models for Plant Disease Detection and Diagnosis.” Computers and Electronics in Agriculture 145: 311–318.

[fsn371348-bib-0010] Gasanov, M. , A. Petrovskaia , A. Nikitin , et al. 2020. “Sensitivity Analysis of Soil Parameters in Crop Model Supported With High‐Throughput Computing.” In International Conference on Computational Science. Springer.

[fsn371348-bib-0011] Gupta, M. , A. Wali , S. Gupta , and S. K. Annepu . 2018. “Nutraceutical Potential of Guava.” In Bioactive Molecules in Food, 1–27. Springer International Publishing.

[fsn371348-bib-0012] Hunter, M. C. , R. G. Smith , M. E. Schipanski , L. W. Atwood , and D. A. Mortensen . 2017. “Agriculture in 2050: Recalibrating Targets for Sustainable Intensification.” Bioscience 67, no. 4: 386–391.

[fsn371348-bib-0013] Jain, R. , P. Singla , R. Sharma , V. Kukreja , and R. Singh . 2023. “Detection of Guava Fruit Disease Through a Unified Deep Learning Approach for Multi‐Classification.” In 2023 IEEE International Conference on Contemporary Computing and Communications (InC4). IEEE.

[fsn371348-bib-0014] Khan, M. A. , M. I. U. Lali , M. Sharif , et al. 2019. “An Optimized Method for Segmentation and Classification of Apple Diseases Based on Strong Correlation and Genetic Algorithm Based Feature Selection.” IEEE Access 7: 46261–46277.

[fsn371348-bib-0015] Kilci, O. , and M. Koklu . 2024. “Classification of Guava Diseases Using Features Extracted From Squeezenet With Adaboost and Gradient Boosting.” In *Proceedings of the 4th International Conference on Frontiers in Academic Research*.

[fsn371348-bib-0016] Merida, M. , and A. Palmateer . 2006. Florida Plant Disease Management Guide: Guava (Psidium guajava), 232. Plant Pathology Department.

[fsn371348-bib-0017] Mirvakhabova, L. , M. Pukalchik , S. Matveev , P. Tregubova , and I. Oseledets . 2018. “Field Heterogeneity Detection Based on the Modified FastICA RGB‐Image Processing.” In Journal of Physics: Conference Series. IOP Publishing.

[fsn371348-bib-0018] Mohanty, S. P. , D. P. Hughes , and M. Salathé . 2016. “Using Deep Learning for Image‐Based Plant Disease Detection.” Frontiers in Plant Science 7: 215232. 10.3389/fpls.2016.01419 PMC5032846 27713752

[fsn371348-bib-0019] Mostafa, A. M. , S. A. Kumar , T. Meraj , H. T. Rauf , A. A. Alnuaim , and M. A. Alkhayyal . 2021. “Guava Disease Detection Using Deep Convolutional Neural Networks: A Case Study of Guava Plants.” Applied Sciences 12, no. 1: 239.

[fsn371348-bib-0020] Murtaza, G. , L. Shuib , A. W. A. Wahab , G. Mujtaba , and G. Raza . 2020. “Ensembled Deep Convolution Neural Network‐Based Breast Cancer Classification With Misclassification Reduction Algorithms.” Multimedia Tools and Applications 79: 18447–18479.

[fsn371348-bib-0021] Saleem, M. H. , J. Potgieter , and K. M. Arif . 2019. “Plant Disease Detection and Classification by Deep Learning.” Plants 8, no. 11: 468. 31683734 10.3390/plants8110468 PMC6918394

[fsn371348-bib-0022] Shadrin, D. , M. Pukalchik , A. Uryasheva , et al. 2020. “Hyper‐Spectral NIR and MIR Data and Optimal Wavebands for Detection of Apple Tree Diseases.” arXiv preprint arXiv:2004.02325.

[fsn371348-bib-0023] Thangaraj, R. , M. Mohan , M. Moulik , A. Logeshwari , T. Loganathan , and P. Koushika . 2023. “A Comparative Study of Deep Learning Models for Guava Leaf Disease Detection.” In 2023 Third International Conference on Advances in Electrical, Computing, Communication and Sustainable Technologies (ICAECT). IEEE.

[fsn371348-bib-0024] Tharwat, A. 2021. “Classification Assessment Methods.” Applied Computing and Informatics 17, no. 1: 168–192.

[fsn371348-bib-0025] Thilagavathi, M. , and S. Abirami . 2017. “Application of Image Processing in Diagnosing Guava Leaf Diseases.” International Journal of Scientific Research and Management (IJSRM) 5, no. 7: 5927–5933.

[fsn371348-bib-0026] Tripathi, P. , N. Kumar , M. Rai , P. K. Shukla , and K. N. Verma . 2023. “Applications of Machine Learning in Agriculture.” In Smart Village Infrastructure and Sustainable Rural Communities, 99–118. IGI Global.

[fsn371348-bib-0028] Wang, G. , Y. Sun , and J. Wang . 2017. “Automatic Image‐Based Plant Disease Severity Estimation Using Deep Learning.” Computational Intelligence and Neuroscience 2017, no. 1: 2917536. 28757863 10.1155/2017/2917536 PMC5516765

[fsn371348-bib-0029] World Scientific and Engineering Academy and Society (WSEAS) . 2004. WSEAS Transactions on Information Science and Applications. WSEAS.

