# Correction to “Automated Guava Disease Detection Using Transfer Learning With ResNet‐101”

**DOI:** 10.1002/fsn3.71460

**Published:** 2026-01-18

Ahmed, M., F. Ahmed, N. S. Naz, et al. 2025. “Automated Guava Disease Detection Using Transfer Learning With ResNet‐101.” *Food Science & Nutrition* 13, no. 12: e71348. https://doi.org/10.1002/fsn3.71348.

In the originally published version of the article, affiliation 4, Acknowledgments and Funding sections are incorrect. The correct information is as follows:

Affiliation 4:

Department of Information Systems, College of Computer and Information Sciences, Princess Nourah bint Abdulrahman University, Riyadh, Saudi Arabia

## Funding

This study was funded by Princess Nourah bint Abdulrahman University Researchers Supporting Project number (PNURSP2025R759), Princess Nourah bint Abdulrahman University, Riyadh, Saudi Arabia. The authors extend their appreciation to the Deanship of Research and Graduate Studies at King Khalid University for funding this work through Large Research Project under grant number RGP2/585/45.

We apologize for this error.

